# Supplementary material for: Sociogenetic Organization of the Red Honey Ant (Melophorus bagoti)
Source: Insects. 2020 Nov 4;11(11):755. doi: 10.3390/insects11110755 (PMC7693516; doi:10.3390/insects11110755)
Supplement: Supplementary file 1 [file insects-11-00755-s001.zip › insects-974356-supplementary materials-1103/insects-974356-TableS1 and TableS2.pdf]

Table S1. Sampling coordinates of the 13 study colonies of *M. bagoti*.

**Colony**	**Latitude**	**Longitude**
CAT1	−23.759016	133.88361
CAT2	−23.756415	133.883348
CAT3	−23.756764	133.88329
CAT4	−23.758111	133.88374
CAT5	−23.759468	133.884414
CAT6	−23.759655	133.884833
CAT7	−23.760535	133.883073
SPR1	−23.84386	133.955756
SPR2	−23.84452	133.956149
SPR3	−23.84431	133.956431
SPR5	−23.84445	133.956707
SPR6	−23.84421	133.956433
SPR7	−23.844121	133.956127

Table S2. Microsatellite markers used in the genetic analyses of *M. bagoti*. For each marker, the following are indicated: the unit of repetition, the primer sequence, the mix in which the markers were amplified, the fluorochrome, the annealing temperature (°C), the size of the PCR product (bp), the range of allele size (bp), the number of alleles, the frequency of the most common allele, the mean observed heterozygosity H_O, and the mean expected heterozygosity H_E. Data are based on the field worker genotypes.

Locus	Unit of Repetition	Primer Sequence (5'->3')	Mix	Fluorochrome	Annealing Temperature (°C)	PCR Product Size (bp)	Range of Allele Size (bp)	Number of Alleles	Frequency of Most Common Allele	HO	HE
Mb06	(AG)14	CGGTGCCGTCATTATATGTCG<br>TACGTGCATCGGTTCAGCAT	1	PET	60	125	138–197	15	0.18	0.83	0.89
Mb07	(AG)16	ATCTGACCGGCGCAATAGTT<br>GAATCACTGTTACCGCCGCT	1	VIC	60	133	149–208	21	0.29	0.90	0.87
Mb08	(AG)10	CCTGGGACGCGGTGTATATG<br>ACGCTTCAGCACGTTCTACC	1	NED	60	138	151–246	25	0.24	0.96	0.91
Mb11	(AG)10	ATGTCGATACACGTGCACGA<br>CCGAAAGTTTCTCCCGGGTA	2	VIC	60	143	155–165	5	0.55	0.57	0.58
Mb13	(AT)12	TTGTCAGCGTGTGGGTAGTC<br>CGGTTACTCGTGATCCTCGG	2	FAM	60	148	158–194	17	0.36	0.84	0.84
Mb15	(AC)8	TCGGAGTAATGGGCATTGCA<br>TTCGACGGATTCCTAGGCTC	3	VIC	59	155	167–175	5	0.45	0.71	0.66
Mb19	(AG)22	TCATGCCTGAAACAGTCGGA<br>CCATCGGTCGTGGTCATTCA	3	PET	60	166	167–212	12	0.21	0.93	0.88
Mb21	(AC)14	GGGTACGAGTTTCATCGATGC<br>CAGCCGAATCAGGTTCCCAA	1	FAM	60	171	241–316	21	0.24	0.90	0.88
Mb24	(AG)16	AATGTACTCGGTGCGCGAAT<br>TAAGATCGGCAGATTGCGCA	2	NED	60	182	198–223	14	0.17	0.92	0.90
Mb28	(AG)22	TTGCACATCCCGTGGATACA<br>TTGTGTCTGCCTTCTGCGAA	3	NED	60	197	195–248	22	0.22	0.91	0.89
Mb30	(AG)23	AGGTCAAGAGTTTGCTCGGT<br>TCTGGAGCGCGACTAATTGG	2	PET	60	211	215–308	27	0.14	0.93	0.94
Mb32	(AG)24	ACATTTCTCGCAGTCTGGCA<br>AGGGACCTGGCGGAGAATAA	3	VIC	60	218	220–285	29	0.14	0.95	0.95
